# Evidence of Exposure to Highly Pathogenic Avian Influenza H5 Viruses in East Greenland Polar Bears

**DOI:** 10.3201/eid3211.261050

**Published:** 2026-11

**Authors:** Kayla J. Buhler, Charlene Ranadheera, Nathalie Bastien, Marcy Zirino, Emily Jenkins, Morten Tryland, Rune Dietz, Jens Søndergaard, Christian Sonne

**Affiliations:** University of Inland Norway, Koppang, Norway (K.J. Buhler, M. Tryland); Cornell University, Ithaca, New York, USA (K.J. Buhler); Public Health Agency of Canada, Winnipeg, Manitoba, Canada (C. Ranadheera, N. Bastien, M. Zirino); University of Saskatchewan, Saskatoon, Saskatchewan, Canada (E. Jenkins); Aarhus University, Arctic Research Cluster, Roskilde, Denmark (R. Dietz, J. Søndergaard, C. Sonne)

**Keywords:** influenza A virus, viruses, respiratory infections, migration, Arctic, bird flu, highly pathogenic avian influenza, HPAI, zoonoses, Greenland

## Abstract

We detected influenza A virus antibodies in plasma collected from East Greenland polar bears during subsistence hunts in 2024 (3/14 [21%]) and 2025 (6/14 [43%]). Four bears from 2025 had antibodies for the globally emerging highly pathogenic avian influenza virus H5 clade 2.3.4.4b, consistent with survival after exposure.

Highly pathogenic avian influenza (HPAI) is an emerging ecologic and public health threat in the Arctic (*1*). Seasonal bird migrations to Arctic nesting grounds create a hot spot for HPAI outbreaks, amplifying interspecies interactions and the risk for viral reassortment and dissemination across migratory flyways (*1*). Recent detection of HPAI in wild Arctic mammals, along with their nutritional and cultural relevance for Indigenous peoples, emphasizes a strong need for surveillance in Arctic ecosystems (*2*–*4*).

Polar bears (*Ursus maritimus*) are Arctic apex predators that face increased challenges associated with their ecologic position at the interface between marine and terrestrial ecosystems. Climate warming and the subsequent loss of sea ice leads to increased time spent on land and exposure to terrestrial pathogens through the exploitation of new food sources (*5*). Cases of deceased polar bears infected with HPAI have been reported in Alaska, USA (H5N1 in 2023), and Svalbard, Norway (H5N5 in 2026), emphasizing that these emerging viruses may pose a risk to Arctic species (*2*,*6*).

As part of an ongoing collaboration with Indigenous hunters, heart blood was collected during subsistence harvests from polar bears that appeared healthy near Scoresby Sound (Ittoqqortoormiit) in East Greenland (Appendix Figure) during the hunting season (January–May). We screened 109 plasma samples (collected during 2016–2025) for antibodies against all influenza A viruses by using a competitive ELISA (cELISA) that targets the influenza A virus (IAV) nucleoprotein (ID Screen Influenza A Antibody Competition Multi-Species; Innovative Diagnostics, https://www.innovative-diagnostics.com), according to the manufacturer’s instructions. We detected no IAV antibodies before 2024. Three bears (all male) harvested during 2024 had signal-to-noise values within the positive range (<45%) of the cELISA (21% [95% CI 8%–48%]; n = 3/14); the number increased to 6 bears (5 male, 1 female) in 2025 (43% [95% CI 21%–67%]; n = 6/14) (Appendix Table).

We conducted further screening on positive samples (n = 9) and a subset of negative samples (n = 3) by using a hemagglutination inhibition assay that used horse red blood cells (Rockland Immunochemicals, https://www.rockland.com), as described by Kayali et al. (*7*), with minor modifications. In brief, we heat-inactivated plasma at 56 °C for 30 minutes, then hem-adsorbed it and treated it with receptor-destroying enzyme. We used avian influenza viruses representative of H5 and H7 subtypes in the assay (Table). Plasma from 4 animals harvested in 2025 that were positive for IAV antibodies on the cELISA were positive (>1:40) for antibodies to H5N1 viruses falling within clade 2.3.4.4b on the hemagglutination inhibition assay, suggesting that the animals had been exposed to an HPAI virus in this clade (Table).

**Table:** Antibodies detected for influenza A viruses and H5 viruses in 12 polar bears sampled in the Ittoqqortoormiit region of East Greenland during 2024–2025 and submitted to the Canada’s National Microbiology Laboratory for screening*

Bear ID	Sex	Year	cELISA†	HI assay‡			
				A/British_Columbia/PHL2032/2024 (clade 2.3.4.4b H5)	A/Texas/37/2024 (clade 2.3.4.4b H5)	A/Vietnam/1203/2004 (clade 1 H5)	A/Anhui/01/13 (H7N9)
63931	M	2024	**3**	20	<20	<20	20
63938	M	2024	**8**	<20	<20	<20	<20
63942	M	2024	**32**	<20	<20	<20	<20
67552	M	2025	**10**	**40**	**40**	5	<5
67554	F	2025	**24**	20	10	<5	<5
67555	M	2025	**5**	**160**	**40**	<5	<5
67556	M	2025	**40**	20	10	<5	<5
67559	M	2025	**10**	**80**	**80**	5	<5
67563	M	2025	**31**	**160**	**160**	20	<5
67560	M	2025	98	<5	<5	<5	<5
67561	M	2025	71	10	5	<5	<5
67565	M	2025	96	<5	<5	<5	<5

*Bold font indicates a positive result. cELISA, competitive ELISA; HI, hemagglutination inhibition. †cELISA results (ID Screen Influenza A Antibody Competition Multi-Species; Innovative Diagnostics, https://www.innovative-diagnostics.com) are represented as the signal-to-noise ratio, i.e., (optical density of the sample/average optical density of the negative controls) × 100. ‡HI assay results are represented as the endpoint dilution factor (2-fold serial dilution).

We first detected IAV antibodies in East Greenland polar bears in 2024 and antibodies to the highly pathogenic H5 clade 2.3.4.4b in 2025. H5N5 and H5N1 strains were previously confirmed in sea birds (including *Uria aalge* [H5N1 in 2022], *Larus hyperboreus* [H5N5 in 2023], *Fulmarus glacialis* [H5N5 in 2023 and 2024], and *Larus marinus* [H5N5 in 2024]) from the west coast of Greenland (*8*,*9*), consistent with our findings of evidence of IAV exposure in East Greenland polar bears in 2024. Positive bears probably were exposed during the summer–fall bird migration the year before the bears were harvested, given that migratory birds are absent during the polar bear hunting season. Hunters reported no clinical signs, suggesting that bears can recover from HPAI infection. That finding is consistent with the high prevalence reported in our study and survival of the bears for months after the departure of migratory birds. Although cross-reactivity with low pathogenic H5 subtypes is possible, plasma from all 4 seropositive bears inhibited hemagglutination of viruses within clade 2.3.4.4b (not observed when tested against clade 1) (Table). That finding strongly supports infection with a virus from clade 2.3.4.4b, which is exclusively an HPAI lineage.

HPAI spillover to mammalian hosts typically occurs through ingestion of infected birds or through contaminated environments (*1*,*3*,*10*). Transmission to wild mammals may involve multiple mechanisms, including direct contact through mucosal or nasal secretions, scavenging dead birds or bird eggs or infected mammals, and mother-to-offspring transmission through lactation (Figure) (*10*). Further studies are needed to identify which mechanisms are relevant for polar bears, although the most likely route is by eating dead or infected birds and mammals.

**Figure:**
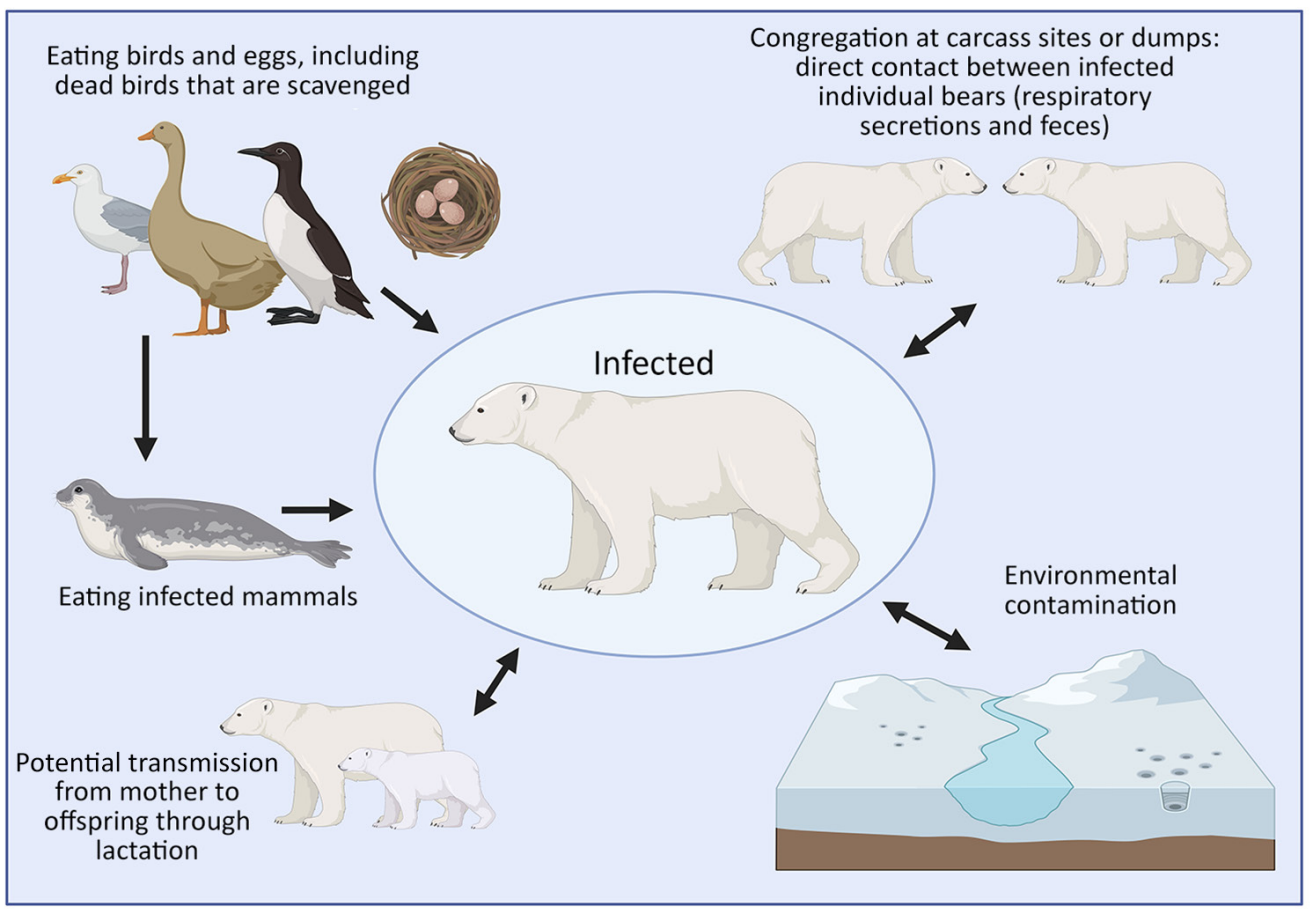
Potential mechanisms of transmission for highly pathogenic avian influenza viruses to polar bears, including direct contact through mucosal or nasal secretions, scavenging dead birds or bird eggs, eating infected mammals, and mother-to-offspring transmission through lactation. Image created by using BioRender (https://www.biorender.com).

Greenland links the East Atlantic and the North American Atlantic flyways (Appendix Figure), and increased surveillance in this region may provide effective early warning signs of HPAI dissemination. Our findings indicate that East Greenland polar bears have been exposed to HPAI in recent years, consistent with the timeline of prior reports of HPAI in birds along the linked migratory superhighways (*3*,*8*,*9*). This carnivore may be an ideal sentinel for emerging HPAI in Arctic ecosystems, given that polar bears opportunistically scavenge coastal nests, birds, and marine mammals as they face nutritional stress associated with sea ice loss (*2*,*5*). Our study also highlights the strength of collaboration between scientists and local subsistence hunters, emphasizing the need for future research that incorporates diverse perspectives and cultural practices to effectively monitor the health of wildlife in these remote regions.

Appendix Additional information about evidence of exposure to highly pathogenic avian influenza H5 viruses in East Greenland polar bears.
