# Supplementary material for: Evidence of Exposure to Highly Pathogenic Avian Influenza H5 Viruses in East Greenland Polar Bears
Source: Emerg Infect Dis. 2026 Nov;32(11):1919–21. doi: 10.3201/eid3211.261050 (PMC13715893; doi:10.3201/eid3211.261050)
Supplement: Supplementary file 1 [file 26-1050-Techapp-s1.pdf]

Article DOI: https://doi.org/10.3201/eid3211.261050

EID cannot ensure accessibility for supplementary materials supplied by authors. Readers who have difficulty accessing supplementary content should contact the authors for assistance.

Evidence of Exposure to Highly Pathogenic Avian Influenza H5 Viruses in East Greenland Polar Bears

Appendix

Appendix Table. Seroprevalence for influenza A viruses detected by competitive ELISA in 109 East Greenland polar bears

Year	2016	2017	2018	2019	2020	2021	2022	2023	2024	2025
Sample size	4	5	12	7	11	12	14	16	14	14
Prevalence, %	0	0	0	0	0	0	0	0	21	43

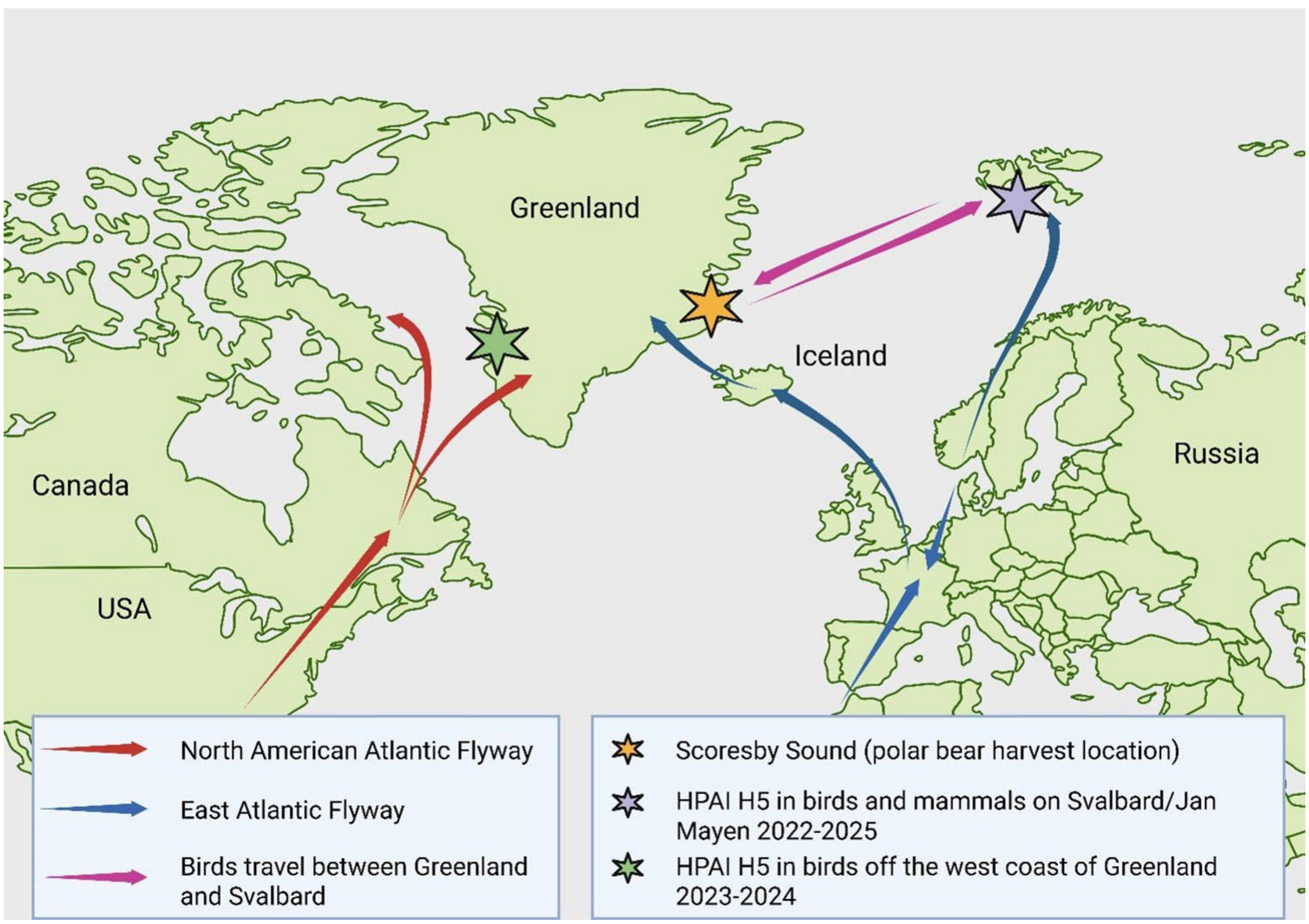

Appendix Figure. Location of polar bear harvests and outbreaks of HPAI H5 along migratory routes of sea and shorebirds during years before the detection of seropositive bears 8,9. The movement of glaucous gulls between Svalbard (where multiple infections with H5 clade 2.3.4.4b have been found in

this species4) and East Greenland is well documented by The Eurasian African Bird Migration Atlas (https://migrationatlas.org). Figure created with biorender.com.
